# Study protocol for a randomized controlled trial with rituximab for psychotic disorder in adults (RCT-Rits)

**DOI:** 10.1186/s12888-023-05250-5

**Published:** 2023-10-23

**Authors:** Susanne Bejerot, Daniel Eklund, Hugo Hesser, Max Albert Hietala, Tarmo Kariis, Niclas Lange, Alexander Lebedev, Scott Montgomery, Axel Nordenskjöld, Predrag Petrovic, Annika Söderbergh, Per Thunberg, Sverre Wikström, Mats B. Humble, RCT-Rits study collaboration group, Peter Asellus, Lise Bergman-Nordgren, Simon Bylund, Jonas Eberhard, Clara Figueras Diaz, Karin Jacobson, Erica Lindeborg, Yvonne Lowert, Erik Nordström, David Terstad Ollén

**Affiliations:** 1 Faculty of Health and Medical Sciences, University Health Care Research Centre, Örebro University, Örebro, Sweden; 2 Faculty of Medicine and Health, School of Medical Sciences, Örebro University, Örebro, Sweden; 3 School of Behavioural, Social and Legal Sciences, Örebro University, Örebro, Sweden; 4 Department of Neurology, Karolinska University Hospital, Stockholm, Sweden; 5 Karlstad Central Hospital, Region Värmland, Karlstad, Sweden; 6 Center for Psychiatry Research (CPF), Center for Cognitive and Computational Neuropsychiatry (CCNP), Department of Clinical Neuroscience, Karolinska Institutet, Stockholm, Sweden; 7 Clinical Epidemiology and Biostatistics, School of Medical Sciences, Faculty of Medicine and Health, Örebro University, Örebro, Sweden; 8 Department of Rheumatology, Örebro University Hospital, Örebro, Sweden; 9 Department of Radiology and Medical Physics, Faculty of Medicine and Health, Örebro University, Örebro, Sweden; 10 Center for Experimental and Biomedical Imaging in Örebro (CEBIO), Faculty of Medicine and Health, Örebro University, Örebro, Sweden; 11 Centre for Clinical Research, County Council of Värmland, Karlstad, Sweden

**Keywords:** Clinical trials, Inflammation, Monoclonal antibodies, Immunology, Magnetic resonance imaging, Schizophrenia & psychotic disorders

## Abstract

**Background:**

The role of inflammation in the aetiology of schizophrenia has gained wide attention and research on the association shows an exponential growth in the last 15 years. Autoimmune diseases and severe infections are risk factors for the later development of schizophrenia, elevated inflammatory markers in childhood or adolescence are associated with a greater risk of schizophrenia in adulthood, individuals with schizophrenia have increased levels of pro-inflammatory cytokines compared to healthy controls, and autoimmune diseases are overrepresented in schizophrenia. However, treatments with anti-inflammatory agents are so far of doubtful clinical relevance.

The primary objective of this study is to test whether the monoclonal antibody rituximab, directed against the B-cell antigen CD20 ameliorates psychotic symptoms in adults with schizophrenia or schizoaffective disorder and to examine potential mechanisms. A secondary objective is to examine characteristics of inflammation-associated psychosis and to identify pre-treatment biochemical characteristics of rituximab responders. A third objective is to interview a subset of patients and informants on their experiences of the trial to obtain insights that rating scales may not capture.

**Methods:**

A proof-of-concept study employing a randomised, parallel-group, double-blind, placebo-controlled design testing the effect of B-cell depletion in patients with psychosis. 120 participants with a diagnosis of schizophrenia spectrum disorders (SSD) (ICD-10 codes F20, F25) will receive either one intravenous infusion of rituximab (1000 mg) or saline. Psychiatric measures and blood samples will be collected at baseline, week 12, and week 24 post-infusion. Brief assessments will also be made in weeks 2 and 7. Neuroimaging and lumbar puncture, both optional, will be performed at baseline and endpoints. Approximately 40 of the patients and their informants will be interviewed for qualitative analyses on the perceived changes in well-being and emotional qualities, in addition to their views on the research.

**Discussion:**

This is the first RCT investigating add-on treatment with rituximab in unselected SSD patients. If the treatment is helpful, it may transform the treatment of patients with psychotic disorders. It may also heighten the awareness of immune-psychiatric disorders and reduce stigma.

**Trial registration:**

NCT05622201, EudraCT-nr 2022–000220-37 version 2.1. registered 14^th^ of October 2022.

**Supplementary Information:**

The online version contains supplementary material available at 10.1186/s12888-023-05250-5.

## Background

### Scientific background and study rationale

Schizophrenia is a major psychiatric disorder, constituting a major source of disability, with extensive consequences for the affected individuals, their relatives and society [1]. Pharmacotherapy based on antipsychotic drugs, all of which block the dopamine-2 (D2) receptors, forms the main specific part of treatment programs. It is established that D2 receptors mediate some key symptoms in the physiopathology of schizophrenia. However, the most burdensome, so-called negative symptoms, do not respond well to D2-blocking treatment. D2 blockers are also linked to several troublesome adverse effects, and there is a selective negative attitude among users to treatment with the present antipsychotic drugs. Treatment resistance is another important issue. Approximately 30% of the patients will not improve much from anti-psychotic drugs. Therefore, it is necessary to investigate drugs with other mechanisms than D2-blockers that may be more effective and accepted by patients with schizophrenia spectrum disorder (SSD). Accordingly, several lines of research try to delineate the pathophysiology of schizophrenia to find other targets, thereby providing novel, more acceptable and effective treatments [2].

#### Inflammation in SSD

Multiple sources of evidence indicate a role of inflammation and immunological mechanisms in schizophrenia [3–8]. Autoimmune diseases and severe infections are risk factors for the later development of schizophrenia [9, 10], elevated inflammatory markers in childhood or adolescence are associated with a greater risk of schizophrenia in adulthood [11, 12], individuals with schizophrenia have increased levels of pro-inflammatory cytokines compared to healthy controls [13–16], and autoimmune diseases are overrepresented in schizophrenia [17]. However, treatments with anti-inflammatory agents are so far of doubtful clinical relevance [18].

There is a strong genetic association between the major histocompatibility complex (MHC) locus and schizophrenia, established by genome-wide association studies (GWAS) [19]. The expression of the genetic loci linked to schizophrenia was enriched by B-lymphocytes involved in acquired immunity (CD19 and CD20 lines), providing strong genetic support for immune dysregulation, specifically implicating B-lymphocytes as a pathogenetic mechanism in schizophrenia. Further research has shown that these associations to a great extent are driven by the complement system [20–23]. This system has a crucial role in synaptic pruning during brain development. Excessive pruning may disable important synaptic connections in the brain, resulting in a vulnerability to neuropsychiatric disorders. In a recent study, complement expression was related to cortical thinning in SSD [22]. Intriguingly, genetic links from schizophrenia to systemic lupus erythematosus (SLE) and rheumatoid arthritis (RA) have been localised to the MHC region, despite divergent clinical manifestations [24, 25]. Interestingly, two cases of bone marrow transplantation further highlight the link between immune genetics and schizophrenia: One case was reported with complete remission from treatment-resistant schizophrenia after a bone marrow transplant [26]. Conversely, another reports the onset of treatment-resistant psychosis after receiving bone marrow from a donor with schizophrenia [27]. Accordingly, immunogenetics strongly supports the immune system’s involvement in schizophrenia pathogenesis and even suggests connections with chronic rheumatological diseases.

Increased plasma protein levels of cytokines and cytokine mRNA expression are widely reported in schizophrenia [13–16]. Of the general proinflammatory cytokines, interleukin-6 (IL-6) is most consistently elevated and has been suggested as a trait marker of schizophrenia. Recently, changes in brain structure were genetically associated with IL-6 by Mendelian randomisation [28], indicating that IL-6 is of central aetiological relevance. Other proinflammatory cytokines, such as IL-1β, TNF-α and IFN-γ are also elevated in most studies. The anti-inflammatory cytokines IL-10 and TGF-β are also mostly elevated, indicating a dysregulated state of the immune system. Other cytokines reported as elevated or deviating in schizophrenia are IL-1 receptor antagonist (IL-1Ra), the soluble receptor of IL-2 (sIL-2R), IL-4, IL-8 and IL-17. Our research group has found similar associations [29]. We compared a mixed sample of markedly ill psychiatric patients (*N* = 39) with participants without psychiatric or somatic disease regarding inflammatory-related markers, using electrochemiluminescence ELISA and real-time qPCR technology. Fourteen of these patients suffered from SSD, and, in this small group, IL-1RA (*p* < 0.001), IL-18 (*p* = 0.006), IL-6 (*p* < 0.002) and TNF-α (*p* = 0.005) protein levels were statically significantly higher compared to matched controls. Also, the mRNA gene expression of *CASP1* (caspase-1, part of the inflammasome complex) was elevated (*p* = 0.008). Other groups have found that inflammation has a closer association with negative symptoms, specifically reward processing and motivational deficits, than with positive symptoms [8]. The roles of astrocytes and regulatory T-cells (Tregs) have also been emphasised [30, 31]. Dysfunctional Tregs have been suggested as a pathophysiological mechanism that links schizophrenia to autoimmune disorders, such as RA and multiple sclerosis (MS). Flow cytometry of immune cells from 40 patients with schizophrenia [32] indicated dysfunctional Tregs and an increased percentage of B-cells (CD19 and CD20) compared to controls. In addition, the percentage of B-cells correlated positively with the severity of psychosis.

The link between an inflammatory activation of the immune system and schizophrenia is well supported, but the chain of causal mechanisms linking a genetic predisposition to the manifestations of schizophrenia is still poorly understood. Autoimmune inflammatory diseases are often characterised by antibodies directed against “self” components, so-called autoantibodies. In autoimmune encephalitides, such antibodies have a pathogenetic relevance, but this mechanism has not been supported in schizophrenia. In other CNS diseases such as MS, however, autoantibodies are not the central pathogenetic mechanism. In MS, T-cell mechanisms and antibody-independent B-cell functions are now considered important pathological processes [33, 34]. The last decade’s advancements in the treatment of this inflammatory CNS disease build on these tenets.

#### Anti-inflammatory treatments in SSD

The main treatment of schizophrenia, antipsychotic drugs, has been shown to exert some anti-inflammatory effects. This is especially pronounced for clozapine [35, 36], the most effective antipsychotic drug hitherto [37], possibly suggesting that clozapine’s immunomodulatory effects are related to its superior efficacy. However, several trials with anti-inflammatory agents, based on the inflammatory hypothesis of schizophrenia, have been published [38–42]. The outcome with these anti-inflammatory augmentations (celecoxib, minocycline, simvastatin, aspirin and prednisolone) has in general been unconvincing, but according to a meta-analysis [18], anti-inflammatory add-on treatment to antipsychotics did show some, albeit clinically modest, improvement in psychotic disorders.

More specific immunomodulatory treatments have recently been tested in SSD. In the first study with tocilizumab, this IL-6-antagonist was not effective [43], possibly due to low IL-6 levels in the study sample. However, in a small study with the TNF-α-antagonist adalimumab as an add-on to risperidone, a significant effect on negative and general symptoms was shown [44]. Several other trials with monoclonal antibodies for SSD are presently ongoing [45, 46], however, apart from our open pilot study [47], rituximab has not been tested in SSD.

#### Rituximab in SSD

Rituximab is a monoclonal antibody that binds to a membrane protein, CD20, which is located on the surface of pre-B cells and mature B lymphocytes. The binding of the antibody to a B cell leads to cytolysis (cell death). Rituximab is a standard treatment for primary B cell type lymphoma, including CNS lymphoma and is included in the treatment guidelines for anti-NMDA receptor encephalitis [48]. It is also a standard therapy for RA and MS. The effect of rituximab on neuroinflammation has clearly been shown by its effect in the treatment of MS [49]. The B cell fills multiple functions in the immune system, including as antigen presenter (activating T cells and other cells), secretion of immunomodulating cytokines, and production of antibodies. By using rituximab as treatment for neurological disorders, the antigen-presenting and immunomodulating functions of the B cells are thought to be largely the property that gives rise to the treatment effect, i.e. not the elimination of the antibodies [34]. The action of rituximab in CNS disorders is thought to be primarily associated with depletion of B cells in the periphery which subsequently leads to decreased B cell entry into the CNS from the bloodstream, i.e. not depending on cytolytic action against B cells within the CNS. CD20 is not expressed on hematopoietic stem cells, pro-B cells (precursor to pre-B cells and B lymphocytes), plasma cells (challenged B lymphocytes producing antibodies) or in normal tissue, which means: 1) the effect of rituximab is reversible and upon completion of treatment, the patient eventually recovers its B cells, which thus regain their function in the immune system, 2) already engineered plasma cells will continue to produce antibodies, and 3) tissues in the body that do not express CD20 are not affected. As rituximab has been used on a long-term basis by thousands of people since the nineties, its side effects in the short and long term are well-known and are usually mild. A dreaded side effect, causing progressive multifocal leukoencephalopathy (PML), has been shown to be very rare, so it does not need to be taken into consideration.

It is hypothesized that the role of the immune system in SSD is mediated by the interaction between B and T cells, glial cells and cytokines. A possible effect of rituximab treatment may be that decreased activation via B cells contributes to the normalization of T-cell-driven inflammation in the brain, possibly through enhancement of T regulatory cells (Tregs) which in turn leads to a normalization of glial activity and eventually a decrease in symptoms.

Between 2019 and 2022, we have run two open pilot trials with a single intravenous dose of rituximab 1000 mg. One study included 9 patients diagnosed with treatment-resistant SSD and the other 10 treatment-resistant patients with obsessive–compulsive disorder (OCD). Results show that seven out of nine SSD patients improved significantly at one or several time points (12, 20 or 40 weeks), but only two of the OCD patients were much improved. Rather few side-effects were reported by the participants. Symptoms such as anxiety and depression were rarely reported [47].

In the present study, we want to test if add-on treatment with rituximab can improve symptoms in patients with SSD in a placebo-controlled setting.

### Proposed study

The purpose of the proposed project is to confirm or reject the hypothesis that adjunctive treatment with rituximab will improve SSD in a proof-of-concept, phase 2, double-blinded, randomised controlled trial (RCT), comparing rituximab and placebo in patients with SSD.

### Study aims and hypotheses

This study aims to determine the safety, tolerability, and efficacy of rituximab as an adjunct to antipsychotic medications in adult patients with schizophrenia or schizoaffective disorder. Based on the promising results of our previous pilot study, we hypothesise that patients with SSD can be successfully treated with this potent immunomodulatory drug.

Our primary hypothesis is that depleting the B-cells with a single intravenous infusion of rituximab in individuals with psychosis will attenuate psychotic symptoms overall and improve function and well-being, relative to placebo. Furthermore, we will explore whether biochemical or clinical characteristics (or combinations thereof) before treatment can predict the response to rituximab treatment.

Finally, patients and their informants will be interviewed separately on their perceptions of changes in participants´ wellbeing and emotional reciprocity in addition to collecting information on their views on the research.

## Methods

This protocol has been prepared in accordance with the Standard Protocol Items: Recommendations for Interventional Trials (SPIRIT) 2013 statement, (see checklist in Additional file 1) [50]. The EudraCT-nr is 2022–000220-37 and the 3.1 version was approved on the 8^th^ of August 2023. Three revisions of the original protocol were performed and accepted by the Ethical Committee on the 7^th^ of Nov 2022, the 2^nd^ of April 2023, and the 30^th^ of August 2023. The primary reason for the first revision was the addition of fMRI scans, for the second revision was changes in title and age group and the addition of qualitative interview, drug screen, assessment for negative symptoms and additional blood tests. Primary reasons for the third revision were removing change in PANSS scores as a measure of response (however it is still the primary outcome measure), the addition of a neurodevelopmental symptoms assessment and another measure for negative symptoms.

We included the first participant at our site, an academic hospital in Örebro on March 22, 2023, and endpoint I was reached for this participant on 22^nd^ of June 2023. Nine additional participants have been included hitherto (1^st^ of September 2023) in Örebro. However, all revisions to the Ethical committee were sought before any participant had reached endpoint I.

We expect recruitment to begin at additional sites during 2023 and 2024. The planned end date is June 30, 2026.

### Response criteria

Response is defined as a CGI-Impovement score of 1 or 2 corresponding to very much improved, assessed or much improved by the investigator.

### Patient and public involvement

The qualitative interviews with patients and informants that are included in this study protocol were partly prepared in collaboration with individuals with lived experience of psychosis.

### Study design and sample

This is a proof-of-concept study. We will conduct a multicentre, placebo-controlled, double-blinded parallel-group, add-on intervention study for patients with SSD, either with stable chronic or acute relapse chronic course, mostly corresponding to "moderately” to “markedly ill” schizophrenia.

### Population

**Fig. 1:**
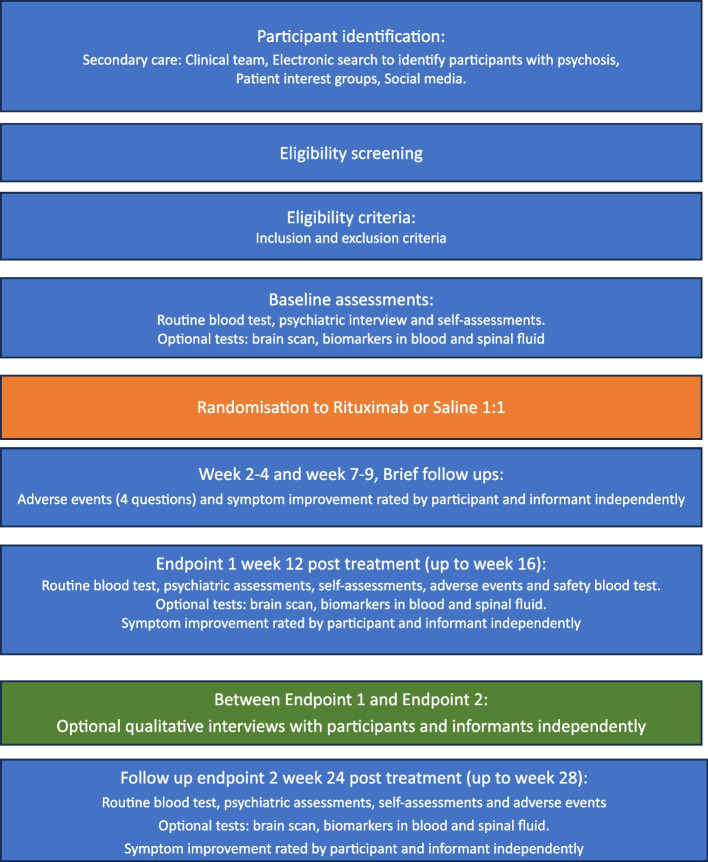
Overview of study design

We will recruit adult patients (age 18–55) (*n* = 120) with *Schizophrenia,* corresponding to ICD-10 code F20, *or Schizoaffective disorder,* corresponding to ICD-10 code F25. Prior to inclusion, participants may have been diagnosed with other psychotic disorders such as *Unspecified nonorganic psychosis* (F29) or *Acute and transient psychotic disorders* (F23). If the patient has had at least one recurrent psychotic episode within the last three months, he/she can be included in the study, given that the diagnostic criteria for schizophrenia or schizoaffective disorder are fulfilled. Patients with schizotypal or delusional disorders (F21 and F22) are not included in this study. The diagnosis will be confirmed with the psychosis section in the M.I.N.I. interview. Patients must be on a stable treatment regime with no recent (within 4 weeks) initiation, cessation or change in antipsychotic medication and remain on their maintenance psychiatric treatment throughout the study. Eligibility criteria are shown in Table 1 and Overview of study design is shown in Fig. 1.

**Table 1:** Eligibility criteria for the RCT-R study

**Inclusion criteria**
• provide informed consent
• willing to consent to blood sampling
• patient aged 18 to 55 years
• duration of illness exceeding 1 year
• diagnosed with schizophrenia spectrum disorder (SSD)
• if female and with any risk for pregnancy, willing to use contraceptives or abstinence if normal and preferred lifestyle
• subjects should be judged by the investigator to be lucid and oriented to person, place, time, and situation when giving the informed consent
• insufficiently recovered from previous antipsychotic treatments
• a minimum score of 4 in CGI-S at baseline
**Exclusion criteria**
• pregnancy or breast-feeding
• weight below 40 kg
• clinically relevant ongoing infection at the discretion of the physician
• chronic infections
• positive test for hepatitis B, hepatitis C, HIV, or TB prior to treatment
• malignancy currently or within 2 years prior to inclusion
• current severe heart failure (NYHA grade IV) or any other severe heart disease (e.g. history of cardiac arrhythmia or myocardial infarction)
• any change of antipsychotic medication within the previous 4 weeks
• unable to make an informed decision to consent to the trial
• ongoing clozapine treatment
• ongoing immunomodulatory treatment
• treatments with monoclonal antibodies within 1 year before the inclusion

Patients are assessed at five time-points; week 0, 2, 7, 12 (endpoint I) and 24 (endpoint II), as outlined in Table 2.

**Table 2:** Outline of the RCT-RITS study

***Week***		**-4 – 0**	**0**	2 (+ 2)	7 (+ 2)	12 (+ 4)	24 (+ 4)
***Visit***		**1**	**2**	3	4	5	6
***Activity/ Assessment number***	**Time to complete**	**Baseline**	**Treatment**	**Check-up**	**Check-up**	**Endpoint I**	**Endpoint II**
**Enrolment** Eligibility screen, Consent form, Demographics, Treatment history, Infection history, Physical examination, Routine blood tests, Immunoglobulins, Test of hepatitis B and C, HIV, and TB. Pregnancy test and Drug screen in urine	40 min	x					
**Intervention:** Rituximab or Saline infusion and auxiliary drugs	4.5 h		x				
**Assessments:** M.I.N.I. interview, psychosis section	10 min	x					
Positive and Negative Syndrome Scale (PANSS), Personal and Social Performance Scale (PSP)	40 min	x				x	x
CGI-S (clinician rated severity)	5 min	x				x	x
CGI-I (clinician rated improvement)	10 min					x	x
CGI-I (informant evaluated improvement)	5 min			x	x	x	x
PGE (patient evaluated improvement)	5 min			x	x	x	x
Patient evaluated health (VAS-health), Level 1 Cross-cutting symptom global symptom severity, MAP-SR	10 min	x				x	x
Adverse Event assessment: open check-up questions	5 min			x	x	x	x
Adverse Event assessment: AAR-R	10 min	x				x	x
Blood test security: leukocyte count/5 part, SR, CRP	10 min	x				x	x
Self-assessment of Negative Symptoms (SNS, patient-rated), Weight	5 min					x	
Blood sample for analyses of PBMC, proteins and gene expression (optional)	15 min	x				x	x
Cerebrospinal fluid tap and fMRI (optional)	40/60 min	x				x	x
Five to Fifteen brief (FTF-brief) filled out by a parent (optional)	5–10 min	x					
Qualitative interview with an informant after 12 weeks (optional)	45–60 min					x	
Qualitative interview with participant after 12 weeks (optional)	45–60 min					x	

*AAR-R* Any Adverse Reactions-Revised, *PBMC* Peripheral blood mononuclear cells, *CGI-I* Clinical Global Impression-Improvement scale, *CGI-S* Clinical Global Impression-Severity scale, *MAP-SR* The Motivation and Pleasure Scale–Self-Report, *PGE* Patient’s Global Evaluation, *TB* tuberculosis

### Intervention

A single intravenous infusion of the monoclonal antibody rituximab (1000 mg) or normal saline will be given to the participants. Rituximab is commercially available and licensed in Sweden for the treatment of RA, lymphoma, granulomatosis with polyangiitis, and pemphigus vulgaris. It is also used off-label for MS, neuromyelitis optica spectrum disorder and autoimmune encephalitis. The approved dosage of rituximab for the treatment of RA or MS is 1000 mg. Clinical improvement in RA is generally seen 16–24 weeks post-treatment [51], but in our pilot study, we noticed a large improvement at week 12 with attenuated effects at week 20. Accordingly, we have chosen endpoint I at week 12 and will follow participants up to week 24 post-infusion.

### Study outcomes

#### Primary outcome

The primary outcome measure is change in symptoms measured as change in Positive and Negative Syndrome Scale (PANSS) score from baseline up to week 12.

#### Secondary outcomes

1. Change in Personal and Social Performance Scale (PSP, measuring overall disability) from baseline up to week 12 and 24.
2. Proportion of responders to treatment, i.e. rated as much or very much improved since baseline according to CGI-I up to week 12 and 24.
3. Improvement since baseline (CGI-I) up to week 12 and 24.
4. Severity according to CGI-S compared to baseline at week 12 and 24.
5. Improvement in PANSS up to week 24 compared to baseline.
6. Differences in patient self-rated health (VAS-health) and PGE since baseline at week 12 and 24.
7. Baseline levels of biochemical markers in relation to treatment response.
8. Safety and tolerability of rituximab during treatment for SSD.
9. Change in brain morphology and/or activity in fMRI.
10. Mental health symptom domains (Level 1 Cross-cutting symptom measure of global symptom severity) in relationship to response.

#### Assessment variables:

1. Positive and Negative Syndrome Scale (PANSS) is a measure of severity in patients with schizophrenia [52, 53]. It is widely used in the study of antipsychotic therapy and is known as the “gold standard” for the assessment of schizophrenia. To assess a patient using PANSS, an approximately 45-min clinical interview is conducted. The patient is rated from 1 to 7 on 30 different symptoms, based on the interview as well as reports from family members or caretaking healthcare workers. The time span considered is the week before the rating. Seven items measure “positive” symptoms, 7 items “negative” symptoms, and 16 items general psychopathology. Each item is graded between 1 and 7. A total score of 30 corresponds to no symptoms, the maximum score is 210 points. ‘Moderately ill’ corresponds to a PANSS of 75, ‘markedly ill’ to a PANSS of 95, and ‘severely ill’ to a PANSS of 116 [54]. We will evaluate item A6 for depression separately at baseline and endpoints in order to assess depressive symptoms. The percentage change of PANSS will be calculated after subtracting the total scores by 30 points (which equals no symptoms) to reach a PANSS 0 score.
2. Personal and Social Performance Scale (PSP) [55] is a 100-point single-item rating scale, subdivided into 10 equal ranges, where 100 represents optimal performance and well-being. The rating is based on assessments of the patient’s functioning in four main areas: 1) socially useful activities, 2) personal and social relationships, 3) self-care, and 4) disturbing and aggressive behaviours.
3. Clinical Global Impression-Improvement scale (CGI-I) measures change in symptoms on a 7-point Likert scale. A score of 1 is very much improved, 2 is much improved, 3 is minimally improved and 4 corresponds to no change, whereas scores of 5, 6 and 7 correspond to deterioration compared to baseline. As with PANSS, the time span considered is the week before the rating. CGI-I will also be administered to the next-of-kin [56].
4. Clinical Global Impression-Severity scale (CGI-S) is a clinician-rated measure of overall clinical severity in the context of the diagnostic group. It is rated on a scale between 1 and 7. A person with no clinical complaints or problems related to the diagnosis (which is most people in a population) will get a score of 1. The score 7, which indicates the highest level of severity, is phrased as “Among the most extremely ill patients”. A score of 2 is borderline ill, 3 is mildly ill, 4 is moderately ill, 5 is markedly ill and 6 is severely ill [56]. In the present study, we expect patients to be moderately ill (or worse) and to improve by at least 1 point at week 12 with rituximab treatment.
5. VAS-health is a patient-evaluated health measure using a horizontal visual analogue scale ranging from 0 (= worst imaginable health) to 10 (= best imaginable health) on the day of the visit. A 25% increase from baseline will be regarded as a self-reported treatment response measure.
6. Patient’s Global Evaluation (PGE) [57] provides a self-administered global measure of improvement on a 7-point Likert scale identical to the CGI-I scoring system.
7. Baseline levels of inflammatory markers in relation to treatment response (CGI-I < 3) will be examined at both endpoints. Changes in inflammatory markers in relation to response will also be investigated.
8. Severity and frequencies of adverse events will be recorded according to Any Adverse Reactions-Revised (AAR-R) [47].
9. Change in brain activity (e.g. blood flow and Default mode network connectivity) and morphology before and after treatment and in relation to response.
10. Level 1 Cross-cutting symptom measure of global symptom severity (CCSM) [58] is a patient-rated measure included in the DSM-5, which assesses mental health domains that are important across psychiatric diagnoses. It includes 13 domains and each of the items is rated 0–4 on a Likert scale. We will investigate for these domains whether endorsement is associated with response.

All clinicians involved in the study will be trained in using PANSS and PSP to reach acceptable agreements between raters.

#### Biomarkers

Levels of biomarkers related to inflammation and glial activation at the start of the study will be compared between the two groups “responders” and “non-responders” in order to identify predictors of treatment response. Profiles of various biomarkers (e.g. cytokine patterns and metabolomes), in addition to absolute values of individual substances, will be assessed to identify biochemical characteristics in patients who benefit from the treatment. Such combinations of biomarkers that can be most informative to distinguish between “responders” and “non-responders” will be analysed with, for example, PCA analysis. Changes in inflammatory markers and other measurements from baseline to week 12 and 24 (Δ values) for all relevant measurements will also be analysed in relation to clinical response after completion of the study.

#### Neuroimaging

Patients will undergo MRI examinations at baseline and endpoints; these procedures are optional. Prior to scanning, patients will be asked to complete a MRI safety checklist. Patients will also be asked if they have taken benzodiazepines and/or hypnotics within 24 h of initiating MRI; those who have will be asked to come back for a later session (more than 24 h later). During scanning, patients will undergo the following protocol:

- High-resolution anatomical MRI scans: Whole-head, T1- and T2 FLAIR-weighted MRI scans, 1-mm isotropic voxels — 6-min scan time.
- Two runs of resting state functional MRI (rsfMRI) scanning – 10 min each. During each rsfMRI, the participants will look at a monitor showing either a crosshair or a low affective naturalistic film. Which alternative (crosshair/movie) that appears first is random.
- Diffusion tensor imaging (DTI): 45 gradient orientations distributed in one shell of b = 1000 plus 6 b0 images; — 6-min scan time.

The total examination time is estimated to be approximately 60 min including surveys and preparations.

Anatomical images will be analysed using voxel-based morphometry (VBM) to see if there are any changes in brain volumes after treatment. DTI and rsfMRI will be used to study potential changes in structural and functional connectivity, respectively. The two rsfMRI acquisitions with different stimuli will be compared and analysed. Recent research suggests that the use of naturalistic film during rsfMRI might be beneficial when measuring changes in neuropsychiatric disorders [59].

#### Qualitative interviews

Participants and informants will be asked to participate in separate qualitative interviews on their subjective experiences of the trial and changes in the participant’s symptoms, 3–5 months post-infusion. Approximately 40 participants and 40 informants will be interviewed. Interviews will be held via telephone or face-to-face. Each interview will last approximately 45–60 min.

The interview guides are semi-structured and include 15 or 19 questions respectively (Additional file 2). The interviews include questions on experience after treatment (e.g. if there are changes in psychotic symptoms, behaviour, motivation and emotions). In addition, four open questions on how informants have experienced the research project will be administered.

Informed consent for this auxiliary study is obtained at baseline. The qualitative data will be analysed through thematic analysis [60]. Participants, informants, and researchers are blinded to treatment allocation.

### Sample size and statistical power

#### Sample size calculation

Different studies report vast variations in placebo response in randomized placebo-controlled trials on schizophrenia, which makes sample size calculation difficult to estimate. The placebo response has increased considerably in more recent studies [61, 62].

An important factor that may affect the placebo response is the selection of patients. If inclusion criteria demand patients to be in relapse or in an acute exacerbation, the natural course of the disease predisposes to regression to the mean, which comes out as a placebo response [61]. We will mostly include chronic stable patients with schizophrenia, which should imply a lower risk for placebo response.

Drop-out rates are also expected to be low, as we use few assessments, only administer one single dose of the research drug and offer a reimbursement (700:- SEK) at both endpoints.

A mean baseline PANSS score of approximately 85 (SD = 17) is anticipated, based on the proposed selection of participants with mostly stable chronic course of their disease.

#### Primary outcome

We expect a mean reduction of 4 points in the PANSS score in the placebo group and a reduction of 14 in the rituximab treatment group, based on the expected low placebo response in this cohort and our results from the pilot study, resulting in endpoint scores of 81 and 71, respectively. With a SD of 17 in both groups and a 10-point difference between groups, an effect size Cohen’s *d* of 0.59 is expected. With an estimated dropout of 10%, a sample size of 51 in each group is needed. The calculations are based on two-sided tests with a power (1 – risk of making a type II error) of 80% and a significance level (risk of type I error) of 5%.

#### Secondary (categorical) outcome

We expect 33% in the rituximab-treated group to be much or very much improved (i.e., score 1 or 2 on CGI-I), compared to an expected 10% in the placebo group. To reach a power of 80% with a significance level of 5% (two-sided), we need to include 47 participants in each group. To allow for a 10% drop-out, we need 52 (47/0.9) participants per group.

Response criterion is defined as much or very much improved according to CGI-I.

#### Sample size

We aim to include a total of 120 participants to account for a loss of degrees of freedom when statistically adjusting for prognostic factors in the model.

### Randomisation and blinding

At baseline, the participants will be randomised to rituximab (10 mg/ml) 1000 mg (100 ml) in 400 ml saline (one single dose) or placebo control (500 ml saline). The administration of rituximab and placebo will be concealed from the research group. Clinicians and researchers will be kept blinded to treatment allocation throughout the study. Treatments will be administered in Region Örebro, University Hospital of Umeå, Karolinska Hospital, Solna and at a local care unit in Helsingborg, where rituximab can be administered to patients by trained personnel. The infusion bag will be masked at the time of preparation and administered via a masked infusion set to maintain blinding.

Randomisation lists are provided with the assistance of the Clinical epidemiology and biostatistics unit, Region Örebro. The randomisation will be done sequentially, due to interim analysis. The first 32 patients will be randomised 1:1 rituximab or placebo, using block-randomisation (i.e. blocks of 2, 4, or 6). After the inclusion of 32 participants, the second block randomisation 1:1 for another 32 patients will be implemented. Similarly, after the inclusion of 64 patients a third block randomisation will be utilised provided that there is no need to terminate the study prematurely.

### Statistical analysis

Descriptive statistics for demographics and baseline outcomes will be presented both overall and separately for the two groups; frequency (%) tables for categorical variables and number of observations, means, standard deviations, medians, quartiles and minimum and maximum values for continuous variables. All applicable statistical tests will be 2-sided and will be performed using a 5% significance level. No multiplicity adjustments will be undertaken as there is only one primary outcome and endpoint. When appropriate, effect size/point estimate, 95% CI, and *p*-values will be presented.

The Primary outcome will be analyzed for the intention to treat (ITT) sample, which is defined as all randomised subjects. General Linear Models will be utilized for comparison of intervention groups at the primary endpoint (week 12 post-infusion). Models will adjust for baseline measurements of the outcome variable PANSS (week 0) and prognostic factors such as Episodic versus chronic; Treatment resistance, yes/no; Schizo-obsessive features, yes/no; Primarily negative symptoms [63, 64], yes/no; Signs of depression yes/no; Neurodevelopmental symptoms in childhood [65] yes/no; Illicit drug use, yes/no; Age at onset < 13 years, 13–17 years, > 17 years; Duration of psychosis; Severity according to CGI-S; Age and sex, in addition to biomarkers.

Descriptive statistics will be used to evaluate if there are any demographic differences between the missing data stratum and the complete data stratum. Under the Missing at Random assumption, missing data will be handled by either Multiple Imputation or Full Information Maximum Likelihood estimation, assuming that missingness is conditional on variables included in the model.

Non-normality robust, equivalent versions of the tests will be performed in the presence of extreme values or violated parametric assumptions. Outliers will be identified through inspection of residual plots and Cook’s distance. Sensitivity analyses will be performed to evaluate the impact of center and prognostic factors on the results by including the interaction terms with intervention groping variable in the model. A sensitivity analysis will also be performed in which center is included as a random effect in the model.

The analysis of the Secondary outcomes (PSP, CGI-S, CGI-I, VAS-health) are of a supportive nature and will not be adjusted for multiplicity. Appropriate tests (depending on the type of data) such as General Linear Models (continuous or pseudo-continuous) or (Ordered) Logistic Regression (binary) will be used for comparisons between intervention groups at endpoints (week 12 and week 24).

For research projects outside the scope of the present preregistration, additional independent statistical analysis plans will be developed and registered at the Open Science Framework before commencing any analyses.

An overview of study procedures is presented in Fig. 1 and all study measures are detailed in Table 2. Recruitment will take place at psychiatric clinics across Sweden, e.g. in Örebro, Stockholm, Karlstad etc. Approximately 14 sites will be involved in the study.

### Participant identification

Potential participants with psychosis will be recruited from psychiatric departments at several sites in Sweden. The central site is Örebro with recruitment within Region Örebro county. We will also inform patient interest associations for psychiatric disorders, outpatient clinics for psychosis and housing for psychiatric patients with chronic SSD about the study. Potential participants with an interest in participating will be interviewed by the local PI to confirm their eligibility to participate. If deemed eligible, participants will be invited to an appointment to complete a full eligibility assessment.

### Eligibility assessment

Assessments will be carried out by a clinician/PI to establish eligibility and to obtain informed consent. Patients will be assessed with the psychosis section of the Mini International Neuropsychiatric Interview (M.I.N.I.) to confirm the ICD-10 diagnosis of schizophrenia and related psychoses. Diagnoses will also be confirmed through medical records and/or information from the patient’s regular psychiatrist. An MRI screening questionnaire will be administered to those willing to give informed consent for neuroimaging.

### Baseline assessment

Demographic background data (including drug use and comorbidities) and treatment history including questions on obsessive–compulsive disorder will be collected from interviews, rating scales and medical records at baseline Assessments will be made with established and validated rating scales and questionnaires (see list of all study measures in Table 3).

**Table 3:** Study measures in the RCT-Rits study

Name of scale	References	Source	Validated	Time of assessment
AAR-R = Any Adverse Reactions-Revised	(Bejerot et al. 2023) [47]	Interviewer assessed	no	Baseline Endpoint I Endpoint II
CGI-S = Clinical Global Impression-Severity scale	(Guy, 1976) [56]	Interviewer assessed	yes	Baseline Endpoint I Endpoint II
CGI-I = Clinical Global Impression-Improvement scale	(Guy, 1976) [56]	Interviewer assessed	yes	Endpoint I Endpoint II
CGI-I = Clinical Global Impression-Improvement scale	(Guy, 1976) [56]	Informant-report	no	Week 2 Week 7 Endpoint I Endpoint II
FTF-Brief = Five to Fifteen – Brief rating scale	(Lugnegård & Bejerot 2019) [65]	Informant-report	yes	Between week 12 and 24
Level 1 Cross-cutting symptom measure of global symptom severity (CCSM)	(APA, 2013) [58]	Self-report	yes	Baseline Endpoint I Endpoint II
MAP-SR = The Motivation and Pleasure – Self Report	(Llerena et al. 2013) [63]	Self-report	yes	Baseline Endpoint I Endpoint II
PANSS = Positive and Negative Syndrome Scale	(Kay et al. 1987) [52]	Interviewer assessed	yes	Baseline Endpoint I Endpoint II
PGE = Patient’s Global Evaluation (of severity improvement)	(Zohar & Judge, 1996) [57]	Self-report	no	Week 2 Week 7 Endpoint I Endpoint II
Adverse events, open check-up questions		Interviewer assessed	no	Week 2 Week 7 Endpoint I Endpoint II
PSP = Personal and Social Performance Scale	(Morosini et al. 2000) [55]	Interviewer assessed	yes	Baseline Endpoint I Endpoint II
SNS = Self-assessment of Negative Symptoms	(Dollfus et al. 2016) [64]	Self-report	yes	Endpoint I
VAS health = Visual Analogue Scale for measuring wellbeing		Self-report	no	Baseline Endpoint I Endpoint II
Qualitative interview (optional)		Interviewer assessed		Between week 12 and 24

All participants will attend a baseline assessment comprising psychiatric measures, blood sampling, lumbar puncture (optional) and neuroimaging (optional). Patients will undergo tests to establish safety/eligibility to receive rituximab, including blood tests to exclude pregnancy and certain infections, including tuberculosis (TB), human immunodeficiency viruses (HIV), and Hepatitis B and C. Participants will be randomised at the time of the infusion by means of sequentially numbered sealed envelopes.

### Intervention

Two hours prior to the infusion and prior to randomisation, all participants are prescribed orally 6 mg betamethasone, 1000 mg paracetamol and 10 mg desloratadine. One single intravenous infusion of rituximab or saline will be given by trained clinical staff under the supervision of a nurse and physician on duty. The infusion takes approximately 4 h. Participants can return home after the end of the infusion.

### Follow-up assessments

Follow-up assessments will take place approximately 2, 7, 12, and 24 weeks post-infusion and will collect data similar to the baseline assessment. Neuroimaging and lumbar puncture (both optional) will be administered only at baseline and endpoints (i.e. week 12 and week 24). If patients are unavailable for follow-up visits at week 2 and 7, these are allowed to be postponed 14 days, and similarly the endpoint assessments for a maximum of 28 days.

## Risk management

### Psychosis-related risks

All patients will continue with their antipsychotic treatment and all patients will be cared for by their regular psychiatric staff. The researcher will stop the assessment if a patient does not wish to continue. Participants may withdraw from the study at any time without giving any reason.

### Somatic-related risks

In our pilot study, one of the patients developed abdominal pain which was reported as a SUSAR (a suspected unexpected serious adverse reaction). After examination by several surgeons, the patient was deemed as having so-called functional symptoms, i.e. no physical disorder could explain the symptoms and they remitted fully. One OCD patient deteriorated compared to her status at inclusion at the one-year follow-up, presumably due to long-term COVID-19 (which a close family member of the patient also developed, which suggests a genetic susceptibility). This patient had contracted COVID-19 prior to inclusion in the study and was fully recovered but relapsed nevertheless after rituximab treatment.

Rituximab is usually well tolerated. More than 10,000 patients are treated yearly in Sweden. In Örebro, rituximab is administered to rheumatoid arthritis patients on a day-ward. The side effects include infusion reactions, skin and mouth reactions, hepatitis B reactivation and in extremely rare cases progressive multifocal leuko-encephalopathy. Since the treatment may activate latent infections, all participants will be screened for hepatitis, tuberculosis, and HIV prior to the study, in order to exclude positive cases. The participants are pre-medicated to reduce the risk of infusion-related side effects (see above).

### Procedure-related risks

#### Risk of infection

Rituximab is associated with an increased risk of infections. In our pilot study, all rituximab patients displayed a clear reduction of circulating B-cells following treatment, from a mean percentage of 3.92 (± 1.31) at baseline to 0.016 (± 0.01) at week 12. However, at 40 weeks after treatment, all patients except two showed signs of repopulation of circulating B-cells.

#### Venepuncture

Venepuncture may be associated with mild discomfort but will be performed by a nurse, trained in venepuncture.

#### Lumbar puncture

Lumbar puncture, which is optional in this study is associated with discomfort in most patients. Lumbar punctures are performed by a physician trained in the procedure. To prevent post-dural puncture headaches, atraumatic, small-bore needles will be used.

#### Neuroimaging

Participants will be provided with a panic button. They can communicate with the researcher and scan operator throughout the MRI scan. Participants are screened prior to examination to ensure that no metal is present on or within the body.

#### Risk to research staff

No risks are anticipated for the staff, but staff will adhere to normal safety procedures.

### Safety considerations for infusion and monitoring of adverse reaction

#### Before infusion

Participants will be selected based on the inclusion and exclusion criteria. Additionally, participants will be tested for Tuberculosis (TB), HIV, and Hepatitis B and C prior to the infusion. Participants are offered vaccination against varicella zoster and COVID, and if accepted vaccinations are given at least 4 weeks prior to infusion. Female participants of childbearing age or who do not practice sexual abstinence will be given a pregnancy test, which must be negative. In addition, they are informed to avoid getting pregnant for 1 year post-infusion and must adhere to using effective contraception during this period, if sexually active.

If a participant show sign of any infection on the day of the planned infusion, the treatment will be postponed.

#### During and after infusion

Infusions will be given at a rheumatology or neurology ward under the supervision of a study nurse. The infusion bag containing rituximab or saline will be covered with a black plastic bag to keep the patient and study nurse blinded. Temperature, cardiac rate and blood pressure will be measured during the infusion, in line with the use of rituximab for treating patients with somatic disorders.

After infusion, participants will be advised to seek help if they feel unwell. They are provided with a card with a telephone number to the responsible clinician. If necessary, we will unblind the participant and inform their health professional whether they received rituximab or normal saline. Post-trial care, if needed, is available within the regular health care system.

### Adverse reactions

Adverse reactions will be recorded at each follow-up visit and reported into the electronic data capture (EDC) platform. Additional safety blood tests will be done at both endpoints (i.e., CRP, SR and 5-part differential haematology).

Adverse events and possible relationship to the study drug will be checked with the following questions at each visit:

- Have you noticed any new symptoms or problems since your last visit? If yes, which?
- Do you believe these symptoms can be related to a possible rituximab treatment?
- Have you noticed if any of your previous side effects have attenuated or increased? If yes, which ones?
- Have you changed your medication since your last visit, if yes which ones and what are the current doses?

Any Adverse Reactions-Revised (AAR-R) is a 26-item questionnaire developed by the research group to identify side effects known to be related to rituximab treatment [47] (Additional file 3). Common side effects of rituximab include headache, fever, chills, stomach pain, nausea, diarrhoea, heartburn, flushing, night sweats, weakness, muscle or joint pain, back pain, or dizziness. These items, and others, are included in the AAR-R questionnaire and for each item severity and frequency are described. An adjusted version of the AAR-R is used to collect baseline data on the expected common side effects.

The sponsor will submit an annual report on the safety. A summary assessment of the safety situation for the subjects and a benefit/risk evaluation will be included.

The annual report will not contain any personal information of study participants.

## Ethics and dissemination

This study was first approved by the Swedish Ethical Review Authority (2022–03827-01) the 10^th^ of August 2022 and the Swedish Medical Products Agency (Eu-nr 2022–000220-37) on the 19^th^ of September 2022 (version 2.1). The study is pre-registered on ClinicalTrials.gov: NCT05622201. For each protocol amendment, ethics approval will be sought before implementing changes to the approved protocol.

### Consent

Participants who meet the inclusion criteria will be asked to participate in this study. They will receive written and verbal information about the study and receive a signed copy of the consent form prior to study entry. Participants will also be requested to give consent that the researchers collect information from an informant (personnel or next-of-kin) on the patient´s symptoms. Separate consents are provided for optional tests and interviews.

A translated consent form is shown in Additional file 4.

### Study management

The study is sponsored by Susanne Bejerot at Region Örebro County, Sweden. She is also the chief investigator and will have overall responsibility for the study. A named principal investigator will take clinical responsibility for study activities at each site. The study has a steering committee, comprising academic and clinical experts in psychiatry, rheumatology, and neuroscience.

All participating clinicians will attend Good Clinical Practice (GCP) courses. Independent monitors will ensure that all data are in accordance with good practice.

Severe adverse outcomes or lack of effect will be assessed to judge whether the trial should be terminated prematurely or not by a Data Monitoring Committee (DMC) independent of the investigators and PIs. The DMC will be unblinded to the data for the interim analyses (i.e. after 32, 64 and 96 participants have reached Endpoint I). The DMC will keep all information confidential except to state if the study should be terminated or proceed. Unblinding is permissible only if considered necessary for the sake of the patient’s health.

### Data management, confidentiality and retention of samples

All potential participants will be assigned a unique study-specific participant identity (ID) number. Anonymised data from assessments will be uploaded to a secure, password-protected database using a secure electronic data capture (EDC) platform (*SMART trial*).

The sampling of biological materials will be done by a standard operating procedure (SOP) used for sampling from other current cohorts. Blood and lumbar puncture samples collected in this study may be stored for up to 10 years after the completion of additional research at the local biobank. Changes in brain morphology and activity will be studied with MRI techniques and data will be stored at a safe place in Örebro University Hospital, Region Örebro and/or Karolinska Hospital. Data collection forms will be stored at a safe place within Örebro University Hospital, Region Örebro. Confidentiality is secured by the use of a study-specific participant ID number. The code list is available for local PIs only and stored at a safe place at each site.

The study is overseen and monitored by the independent Clinical Trial Unit at Region Örebro län, Örebro, Sweden. Each site will be monitored between 3 and 5 times.

### Dissemination plan

The study will be published in peer-reviewed journals and will conform to the guidelines of the International Committee of Medical Journal Editors. Findings will be disseminated to clinicians and researchers through conferences and meetings. In addition, non-specialist audiences (general public) will be reached through press releases, disseminated via Meltwater and the Swedish Research Council service *Expertsvar* to selected media. Different national patient organisations will be approached for popular science presentations. There will be no publication restrictions and professional writers will not be involved.

We will grant full public access to the full protocol, but not to participant-level datasets.

## Discussion

In accordance with the inflammatory hypothesis of psychosis, we will investigate an add-on treatment with one single dose of the target-specific intervention rituximab (anti-CD20 monoclonal antibody) in unselected SSD patients. If the treatment is helpful, it may transform the treatment of patients with psychotic disorders. It may also heighten the awareness of immune-psychiatric disorders and reduce stigma.

By examining the therapeutic potential of targeting CD20-positive lymphocytes in psychosis, this RCT will provide evidence of whether such cells are engaged in the pathophysiology of schizophrenia. Moreover, the use of neuroimaging, peripheral blood biomarkers and cerebrospinal fluid exploration before and after rituximab treatment, may further disentangle potential mechanisms of the putative effect.

The use of qualitative interviews with participants and informants may shed light on treatment-related changes that are difficult to capture with traditional rating scales.

Several considerations have led us to study the repurposing of rituximab for the treatment of schizophrenia. In 1997, rituximab was one of the first monoclonal antibodies approved for the treatment of human diseases. Accordingly, the clinical experience with rituximab in different populations is immense, when compared to more recently developed substances. It is one of the most frequently used drugs in severe rheumatological disorders and other autoimmune states. In 2023, rituximab was included on the World Health Organization’s Essential Medicines List for use in multiple sclerosis due to convincing evidence and despite its off-label status [66]. This corroborates rituximab’s ability to affect CNS targets and thereby its suitability for a study of psychosis treatment. However, due to safety concerns, we have chosen a medium rituximab dose that is not repeated, and we do not combine it with methotrexate (which is recommended in the treatment of RA). While this may imply a risk of insufficient potency of the intended effects, it will decrease the risk of adverse effects for the included participants.

### Supplementary Information

**Additional file 1.** SPIRIT 2013 Checklist: Recommended items to address in a clinical trial protocol and related documents*.

**Additional file 2.** 

**Additional file 3.** Any Adverse Reactions Revised (AAR-R).

**Additional file 4.** Consent to participate in the study.

## Data Availability

The sponsor/PIs involved in the manuscript writing will have access to the final data set on clinical data. Investigators involved in the fMRI assessments and biological specimens will have access to corresponding data. No contractual agreements limit access for investigators.

## Abbreviations

AAR-R: Any Adverse Reactions-Revised
CASP: Caspase
CCSM: DSM-5 Level 1 Cross-Cutting Symptom Measure
CGI-I: Clinical Global Impression-Improvement scale
CGI-S: Clinical Global Impression-Severity scale
CNS: Central nervous system
CRP: C-reactive protein
D2: Dopamine-2
DSM-5: 5Th edition of the Diagnostic and Statistical Manual of Mental Disorders
DTI: Diffusion tensor imaging
EDC: Electronic data capture
GWAS: Genome-wide association studies
ICD-10: International Classification of Diseases 10th Revision
ID: Identity
IFN-γ: Interferon Gamma
IL: Interleukin
ITT: Intention to treat (ITT)
M.I.N.I.: Mini International Neuropsychiatric Interview
MHC: Major histocompatibility complex
MRI: Magnetic resonance imaging
MS: Multiple sclerosis
OCD: Obsessive-compulsive disorder
PANSS: Personal and Social Performance Scale
PBMC: Peripheral blood mononuclear cells
PCA: Principal component analysis
PGE: Patient’s Global Evaluation
PML: Progressive multifocal leukoencephalopathy
PSP: Personal and Social Performance Scale
RA: Rheumatoid arthritis
RCT: Randomised controlled trial
rsfMRI: Resting-state functional MRI
SEK: Swedish crowns
SLE: Systemic lupus erythematosus
SOP: Standard operating procedure
SR: Sedimentation rate
SSD: Schizophrenia spectrum disorders
SUSAR: Suspected unexpected serious adverse reaction
TB: Tuberculosis
TGF: Transforming growth factor
TNF: Tumor necrosis factor
VAS: Visual analogue scale
VBM: Voxel-based morphology

## References

[1] Solmi M, Seitidis G, Mavridis D, Correll CU, Dragioti E, Guimond S, et al. Incidence, prevalence, and global burden of schizophrenia - data, with critical appraisal, from the Global Burden of Disease (GBD) 2019. Mol Psychiatry. 2023. 10.1038/s41380-023-02138-4. 10.1038/s41380-023-02138-4 37500825

[2] Kaar SJ, Natesan S, McCutcheon R, Howes OD (2020). Antipsychotics: Mechanisms underlying clinical response and side-effects and novel treatment approaches based on pathophysiology. Neuropharmacology.

[3] Khandaker GM, Cousins L, Deakin J, Lennox BR, Yolken R, Jones PB (2015). Inflammation and immunity in schizophrenia: implications for pathophysiology and treatment. The Lancet Psychiatry.

[4] Müller N (2018). Inflammation in Schizophrenia: Pathogenetic aspects and therapeutic considerations. Schizophr Bull.

[5] Severance EG, Dickerson FB, Yolken RH (2018). Autoimmune phenotypes in schizophrenia reveal novel treatment targets. Pharmacol Ther.

[6] Miller BJ, Goldsmith DR (2020). Evaluating the Hypothesis That Schizophrenia Is an Inflammatory Disorder. Focus (Am Psychiatr Publ).

[7] Messina A, Concerto C, Rodolico A, Petralia A, Caraci F, Signorelli MS (2023). Is it time for a paradigm shift in the treatment of schizophrenia? The use of inflammation-reducing and neuroprotective drugs - a review. Brain Sci.

[8] Thylur DS, Goldsmith DR (2022). Brick by Brick: Building a Transdiagnostic Understanding of Inflammation in Psychiatry. Harv Rev Psychiatry.

[9] Benros ME, Nielsen PR, Nordentoft M, Eaton WW, Dalton SO, Mortensen PB (2011). Autoimmune diseases and severe infections as risk factors for schizophrenia: a 30-year population-based register study. Am J Psychiatry.

[10] Jeppesen R, Benros ME (2019). Autoimmune Diseases and Psychotic Disorders. Front Psychiatry.

[11] Khandaker GM, Pearson RM, Zammit S, Lewis G, Jones PB (2014). Association of serum interleukin 6 and C-reactive protein in childhood with depression and psychosis in young adult life: a population-based longitudinal study. JAMA Psychiat.

[12] Metcalf SA, Jones PB, Nordstrom T, Timonen M, Mäki P, Miettunen J (2017). Serum C-reactive protein in adolescence and risk of schizophrenia in adulthood: A prospective birth cohort study. Brain Behav Immun.

[13] Miller BJ, Buckley P, Seabolt W, Mellor A, Kirkpatrick B (2011). Meta-analysis of cytokine alterations in schizophrenia: clinical status and antipsychotic effects. Biol Psychiatry.

[14] Pillinger T, Osimo EF, Brugger S, Mondelli V, McCutcheon RA, Howes OD (2019). A Meta-analysis of Immune Parameters, Variability, and Assessment of Modal Distribution in Psychosis and Test of the Immune Subgroup Hypothesis. Schizophr Bull.

[15] Dawidowski B, Górniak A, Podwalski P, Lebiecka Z, Misiak B, Samochowiec J (2021). The Role of Cytokines in the Pathogenesis of Schizophrenia. J Clin Med.

[16] Szabo A, O'Connell KS, Ueland T, Sheikh MA, Agartz I, Andreou D (2022). Increased circulating IL-18 levels in severe mental disorders indicate systemic inflammasome activation. Brain Behav Immun.

[17] Eaton WW, Byrne M, Ewald H, Mors O, Chen CY, Agerbo E, Mortensen PB (2006). Association of schizophrenia and autoimmune diseases: linkage of Danish national registers. Am J Psychiatry.

[18] Jeppesen R, Christensen RHB, Pedersen EMJ, Nordentoft M, Hjorthøj C, Köhler-Forsberg O, Benros ME (2020). Efficacy and safety of anti-inflammatory agents in treatment of psychotic disorders - A comprehensive systematic review and meta-analysis. Brain Behav Immun.

[19] Ripke S (2014). Schizophrenia Working Group of the Psychiatric Genomics Consortium. Biological insights from 108 schizophrenia-associated genetic loci. Nature.

[20] Sekar A, Bialas AR, de Rivera H, Davis A, Hammond TR, Kamitaki N (2016). Schizophrenia risk from complex variation of complement component 4. Nature.

[21] Cooper JD, Ozcan S, Gardner RM, Rustogi N, Wicks S, van Rees GF (2017). Schizophrenia-risk and urban birth are associated with proteomic changes in neonatal dried blood spots. Transl Psychiatry.

[22] Ji E, Boerrigter D, Cai HQ, Lloyd D, Bruggemann J, O'Donnell M (2022). Peripheral complement is increased in schizophrenia and inversely related to cortical thickness. Brain Behav Immun.

[23] Gracias J, Orhan F, Hörbeck E, Holmén-Larsson J, Khanlarkani N, Malwade S (2022). Cerebrospinal fluid concentration of complement component 4A is increased in first episode schizophrenia. Nat Commun.

[24] Kamitaki N, Sekar A, Handsaker RE, de Rivera H, Tooley K, Morris DL (2020). Complement genes contribute sex-biased vulnerability in diverse disorders. Nature.

[25] Zamanpoor M, Ghaedi H, Omrani MD (2020). The genetic basis for the inverse relationship between rheumatoid arthritis and schizophrenia. Mol Genet Genomic Med.

[26] Miyaoka T, Wake R, Hashioka S, Hayashida M, Oh-Nishi A, Azis IA (2017). Remission of Psychosis in Treatment-Resistant Schizophrenia following Bone Marrow Transplantation: A Case Report. Front Psychiatry.

[27] Sommer IE, van Bekkum DW, Klein H, Yolken R, de Witte L, Talamo G (2015). Severe chronic psychosis after allogeneic SCT from a schizophrenic sibling. Bone Marrow Transplant.

[28] Williams JA, Burgess S, Suckling J, Lalousis PA, Batool F, Griffiths SL (2022). Inflammation and Brain Structure in Schizophrenia and Other Neuropsychiatric Disorders: A Mendelian Randomization Study. JAMA Psychiatr.

[29] Hylén U, Eklund D, Humble M, Bartoszek J, Särndahl E, Bejerot S (2020). Increased inflammasome activity in markedly ill psychiatric patients: An explorative study. J Neuroimmunol.

[30] Dietz AG, Goldman SA, Nedergaard M (2020). Glial cells in schizophrenia: a unified hypothesis. Lancet Psychiatry.

[31] Corsi-Zuelli F, Deakin B, de Lima MHF, Qureshi O, Barnes NM, Upthegrove R (2021). T regulatory cells as a potential therapeutic target in psychosis? Current challenges and future perspectives. Brain Behav Immun Health.

[32] Sahbaz C, Zibandey N, Kurtulmus A, Duran Y, Gokalp M, Kırpınar I (2020). Reduced regulatory T cells with increased proinflammatory response in patients with schizophrenia. Psychopharmacol (Berl).

[33] Kunkl M, Frascolla S, Amormino C, Volpe E, Tuosto L (2020). T Helper Cells: The Modulators of Inflammation in Multiple Sclerosis. Cells.

[34] Li R, Patterson KR, Bar-Or A (2018). Reassessing B cell contributions in multiple sclerosis. Nat Immunol.

[35] Leykin I, Mayer R, Shinitzky M (1997). Short and long-term immunosuppressive effects of clozapine and haloperidol. Immunopharmacology.

[36] Giridharan VV, Scaini G, Colpo GD, Doifode T, Pinjari OF, Teixeira AL (2020). Clozapine Prevents Poly (I:C) Induced Inflammation by Modulating NLRP3 Pathway in Microglial Cells. Cells.

[37] Mizuno Y, McCutcheon RA, Brugger SP, Howes OD (2020). Heterogeneity and efficacy of antipsychotic treatment for schizophrenia with or without treatment resistance: a meta-analysis. Neuropsychopharmacology.

[38] Rappard F, Mueller N (2004). Celecoxib add-on therapy does not have beneficial antipsychotic effects over risperidone alone in schizophrenia. Neuropsychopharmacology.

[39] Deakin B, Suckling J, Barnes TRE, Byrne K, Chaudhry IB, Dazzan P (2018). The benefit of minocycline on negative symptoms of schizophrenia in patients with recent-onset psychosis (BeneMin): a randomised, double-blind, placebo-controlled trial. Lancet Psychiatry.

[40] Sommer IE, Gangadin SS, de Witte LD, Koops S, van Baal C, Bahn S (2021). Simvastatin Augmentation for Patients With Early-Phase Schizophrenia-Spectrum Disorders: A Double-Blind, Randomized Placebo-Controlled Trial. Schizophr Bull.

[41] Weiser M, Zamora D, Levi L, Nastas I, Gonen I, Radu P (2021). Adjunctive Aspirin vs Placebo in Patients With Schizophrenia: Results of Two Randomized Controlled Trials. Schizophr Bull.

[42] Nasib LG, Gangadin SS, Rossum IW, Boudewijns ZSRM, de Witte LD, Wilting I (2021). The effect of prednisolone on symptom severity in schizophrenia: A placebo-controlled, randomized controlled trial. Schizophr Res.

[43] Girgis RR, Ciarleglio A, Choo T, Haynes G, Bathon JM, Cremers S (2018). A randomized, double-blind, placebo-controlled clinical trial of tocilizumab, an interleukin-6 receptor antibody for residual symptoms in schizophrenia. Neuropsychopharmacology.

[44] Motamed M, Karimi H, Sanjari Moghaddam H, Taherzadeh Boroujeni S, Sanatian Z, Hasanzadeh A (2022). Risperidone combination therapy with adalimumab for treatment of chronic schizophrenia: a randomized, double-blind, placebo-controlled clinical trial. Int Clin Psychopharmacol.

[45] Chaves CB, Vieira-Coelho MA (2020). Clinical trials with monoclonal antibodies in schizophrenia. Schizophr Res.

[46] Foley ÉM, Griffiths SL, Murray A, Rogers J, Corsi-Zuelli F, Hickinbotham H (2023). Protocol for the Psychosis Immune Mechanism Stratified Medicine (PIMS) trial: a randomised double-blind placebo-controlled trial of single-dose tocilizumab in patients with psychosis. BMJ Open.

[47] Bejerot S, Sigra Stein S, Welin E, Eklund D, Hylén U, Humble MB (2023). Rituximab as an adjunctive treatment for schizophrenia spectrum disorder or obsessive-compulsive disorder: Two open-label pilot studies on treatment-resistant patients. J Psychiatr Res.

[48] Wandinger KP, Saschenbrecker S, Stoecker W, Dalmau J (2011). Anti-NMDA-receptor encephalitis: a severe, multistage, treatable disorder presenting with psychosis. J Neuroimmunol.

[49] Svenningsson A, Frisell T, Burman J, Salzer J, Fink K, Hallberg S (2022). Safety and efficacy of rituximab versus dimethyl fumarate in patients with relapsing-remitting multiple sclerosis or clinically isolated syndrome in Sweden: a rater-blinded, phase 3, randomised controlled trial. Lancet Neurol.

[50] Chan AW, Tetzlaff JM, Altman DG, Laupacis A, Gøtzsche PC, Krleža-Jerić K, et al. SPIRIT 2013 statement: defining standard protocol items for clinical trials. Ann Intern Med. 2013;158:200–7. 10.7326/0003-4819-158-3-201302050-00583 PMC5114123 23295957

[51] FASS. The Swedish Physicians’ Desk Reference https://www.fass.se/LIF/product?nplId=20130208000060&userType=0. retrieved on Aug 18^th^ 2023.

[52] Kay SR, Fiszbein A, Opler LA (1987). The positive and negative syndrome scale (PANSS) for schizophrenia. Schizophr Bull.

[53] Knorring L, Lindström E (1992). The Swedish version of the Positive and Negative Syndrome Scale (PANSS) for schizophrenia. Acta Psychiatr Scand.

[54] Leucht S, Kane JM, Kissling W, Hamann J, Etschel E, Engel RR (2005). What does the PANSS mean?. Schizophrenia Res.

[55] Morosini PL, Magliano L, Brambilla L, Ugolini S, Pioli R (2000). Development, reliability and acceptability of a new version of the DSM-IV Social and Occupational Functioning Assessment Scale (SOFAS) to assess routine social functioning. Acta Psychiatr Scand.

[56] Guy W (1976). ECDEU assessment manual for psychopharmacology - revised.

[57] Zohar J, Judge R (1996). Paroxetine versus clomipramine in the treatment of obsessive-compulsive disorder. OCD Paroxetine Study Investigators. Br J Psychiatry.

[58] Doss RA, Lowmaster SE. Validation of the DSM-5 Level 1 Cross-Cutting Symptom Measure in a Community Sample. Psychiatry Res. 2022;318:114935. 10.1016/j.psychres.2022.114935 36332507

[59] Kringelbach ML, Perl YS, Tagliazucchi E, Deco G (2023). Toward naturalistic neuroscience: Mechanisms underlying the flattening of brain hierarchy in movie-watching compared to rest and task. Sci Adv.

[60] Braun V, Clarke V (2022). Thematic analysis: a practical guide.

[61] Alphs L, Benedetti F, Fleischhacker WW, Kane JM (2012). Placebo-related effects in clinical trials in schizophrenia: what is driving this phenomenon and what can be done to minimize it?. Int J Neuropsychopharmacol.

[62] Brannan SK, Sawchak S, Miller AC, Lieberman JA, Paul SM, Breier A (2021). Muscarinic Cholinergic Receptor Agonist and Peripheral Antagonist for Schizophrenia. N Engl J Med.

[63] Llerena K, Park SG, McCarthy JM, Couture SM, Bennett ME, Blanchard JJ (2013). The Motivation and Pleasure Scale-Self-Report (MAP-SR): reliability and validity of a self-report measure of negative symptoms. Compr Psychiatry.

[64] Dollfus S, Mach C, Morello R (2016). Self-Evaluation of Negative Symptoms: A Novel Tool to Assess Negative Symptoms. Schizophr Bull.

[65] Lugnegård T, Bejerot S (2019). Retrospective parental assessment of childhood neurodevelopmental problems: the use of the Five to Fifteen questionnaire in adults. BJPsych Open.

[66] World Health Organization. The selection and use of essential medicines 2023. Executive Summary of the report of the 24th WHO Expert Committee on Selection and Use of Essential Medicines, Geneva 2023. Retrieved 4^th^ of Sep 2023 from https://list.essentialmeds.org/recommendations/1348.

