# Supplementary material for: Study protocol for a randomized controlled trial with rituximab for psychotic disorder in adults (RCT-Rits)
Source: BMC Psychiatry. 2023 Oct 23;23:771. doi: 10.1186/s12888-023-05250-5 (PMC10594806; doi:10.1186/s12888-023-05250-5)
Supplement: Supplementary file 2 — Additional file 2. [file 12888_2023_5250_MOESM2_ESM.docx]

**Additional file 2**

**A: RCT-rits – Qualitative interview guide, patient**

1. I want to talk to you about your experiences with the study and if your mood has changed in any way. But first, I would like to know what has been helpful for you in your treatment before participating in this study (Medication, which? Counselling? Other interventions?)?

2. Do you remember when you received treatment with rituximab or placebo?
(If necessary, help with time of treatment)

3. Did you have any expectations about the treatment in the study? (If yes: What expectations?)

4. Do you think you received active treatment (rituximab) or placebo? (If the patient answers yes or no: Why do you think so?)

5. Do you remember how you felt during the time period when you joined the study?

6. How do you feel today compared to when you joined the study? (If any change: In what way do you feel better/worse?)

7. Has there been any change in your psychotic symptoms since you joined the study? (If yes: In what way? Can you give any examples?)

8. Has there been any change in your general psychological well-being, for example anxiety symptoms or depression, since you joined the study?

9. Has there been any change in your behavior since you joined the study? (If yes: In what way? Can you give examples?) (Help the patient with examples if necessary)

10. Has there been any change in your energy level, for instance that you feel more tired or energetic, since you joined the study? (If yes: In what way? Can you give examples?)

11. Has there been any change in your motivation, for instance what you want to do in everyday life, since you joined the study? (If yes: In what way? Can you give examples?)

12. Has there been any change in how you feel in your relationships since you joined the study? (If yes: In what way? Can you give examples?)

13. Has there been any change in your emotional life since you joined the study, like your ability to experience and manage different emotions? (If yes: In what way? Can you give examples?)

14. Are there any other changes you have noticed since you joined the study? (If change is experienced: Do you think the changes are due to treatment with rituximab or could it be due to other causes? What causes do you think of then?)

15. a) Should we have done anything differently in the study?

b) Is there something you want to communicate to the researchers in the study?

c) Is there anything you would like to say to other individuals with psychotic illness about the study?

**B: RCT-rits – Qualitative interview guide, informants**

1. What is your relationship with X? (Family/friendship/professional?)

2. How much have you seen X? During what time period?

3. Do you know for how long X has been ill with psychotic illness?

4. Did you have any expectations of the treatment in the study? (If yes: why?)

5. Do you remember when X received the rituximab/placebo treatment in the study? (If needed, help with time indication)

6. How do you think X is feeling now in relation to when X joined the study? (If there is a difference: In what way does X feel better/worse?)

7. Do you think there has been any change in X's behavior since the start of the study? (If yes: In what way? Can you give any examples?)

8. Do you think there has been any change in X's energy level since the start of the study? (If yes: In what way? Can you give any examples?)

9. Do you think there has been any change in X's mental state (mood/anxiety level) since the start of the study? (If yes: In what way? Can you give any examples?)

10. Do you think there has been any change in X's psychotic symptoms since the start of the study? (If yes: In what way? Can you give any examples?)

11. Do you think there has been any change in X's motivation since the start of the study? (If yes: In what way? Can you give any examples?)

12. Do you think there has been any change in X's emotional contact/warmth since the start of the study? (If yes: In what way? How is it expressed? Can you give any examples?)

13. Do you think there has been any change in X's interest in others, or in the outside world? (If yes: In what way? Can you give any examples?)

14. Have you told X about what you perceive as (possible) change/lack of change since the start of the study? (If yes: How did X react to that?)

15. Do you think X would notice/has noticed a possible change? (If yes: In what way is it expressed? Can you give any examples?)

16. If so, have you been able to talk about it? (If yes: In what way? Can you give any examples?)

17. How does it feel to experience/not experience this (if any) change? (If change: Do you think that the possible change is due to receiving active treatment with rituximab or could there be other causes?)

18. What causes do you think of?

19. Do you feel disappointment/positive experience of the change/lack of change?

Additional four questions:

1. Should we have done anything differently in the study/study design?

2. Is there something that you would like to communicate to the researchers in the study?

3. Is there anything you as a relative/friend/staff would like to communicate to other people with psychosis regarding the study?

4. Is there a difference between getting the written questions in advance, instead of getting them directly during the interview?
