# Supplementary material for: Study protocol for a randomized controlled trial with rituximab for psychotic disorder in adults (RCT-Rits)
Source: BMC Psychiatry. 2023 Oct 23;23:771. doi: 10.1186/s12888-023-05250-5 (PMC10594806; doi:10.1186/s12888-023-05250-5)
Supplement: Supplementary file 3 — Additional file 3. Any Adverse Reactions Revised (AAR-R). [file 12888_2023_5250_MOESM3_ESM.docx]

**Additional file 3:** Any Adverse Reactions Revised (AAR-R)

| Date: Visit No: Participant ID: | | | | | | | | | | |
| --- | --- | --- | --- | --- | --- | --- | --- | --- | --- | --- |
| Have you experienced any new symptoms after you received the study drug?  (The interviewer asks about each symptom in the list below and the frequency). | | | | | | | | | | |
| **Symptoms** | **None** | **Mild** | **Moderate** | **Markedly** | **Sparsely occuring** | **Recurrent, daily** | **Ongoing: Yes/No/ Unknown** | **Associated with study drug:**  **Yes/No/ Unknown** | **SAE/SUSAR** | **Comment** |
| *Body as a whole* |  |  |  |  |  |  |  |  |  |  |
| Fever |  |  |  |  |  |  |  |  |  |  |
| Freezing |  |  |  |  |  |  |  |  |  |  |
| Hot flushes |  |  |  |  |  |  |  |  |  |  |
| Sweating |  |  |  |  |  |  |  |  |  |  |
| Erythema |  |  |  |  |  |  |  |  |  |  |
| Itching |  |  |  |  |  |  |  |  |  |  |
| Hives |  |  |  |  |  |  |  |  |  |  |
| Infection |  |  |  |  |  |  |  |  |  |  |
| Fatigue |  |  |  |  |  |  |  |  |  |  |
| Headache |  |  |  |  |  |  |  |  |  |  |
| Abdominal pain |  |  |  |  |  |  |  |  |  |  |
| Backache |  |  |  |  |  |  |  |  |  |  |
| Myalgias |  |  |  |  |  |  |  |  |  |  |
| Arthralgias |  |  |  |  |  |  |  |  |  |  |
| Other pain |  |  |  |  |  |  |  |  |  |  |
| Rhinitis |  |  |  |  |  |  |  |  |  |  |
| Sinusitis |  |  |  |  |  |  |  |  |  |  |
| Pharyngitis |  |  |  |  |  |  |  |  |  |  |
| Cough |  |  |  |  |  |  |  |  |  |  |
| Asthma |  |  |  |  |  |  |  |  |  |  |
| Dyspnoea |  |  |  |  |  |  |  |  |  |  |
| Nausea |  |  |  |  |  |  |  |  |  |  |
| Vomiting |  |  |  |  |  |  |  |  |  |  |
| Diarrhoea |  |  |  |  |  |  |  |  |  |  |
| Unsteadiness |  |  |  |  |  |  |  |  |  |  |
| Anxiety |  |  |  |  |  |  |  |  |  |  |
| Oedema |  |  |  |  |  |  |  |  |  |  |
| Angioedema |  |  |  |  |  |  |  |  |  |  |
| Other |  |  |  |  |  |  |  |  |  |  |
