# Supplementary material for: Study protocol for a randomized controlled trial with rituximab for psychotic disorder in adults (RCT-Rits)
Source: BMC Psychiatry. 2023 Oct 23;23:771. doi: 10.1186/s12888-023-05250-5 (PMC10594806; doi:10.1186/s12888-023-05250-5)
Supplement: Supplementary file 4 — Additional file 4. Consent to participate in the study. [file 12888_2023_5250_MOESM4_ESM.docx]

**Additional file 4:Consent to participate in the study**

I have received oral and written information about the study and have had the opportunity to ask questions. I get to keep the written information.

|  | | Yes | | No | |
| --- | --- | --- | --- | --- | --- |
| - I agree to participate in the study RCT-RITS, Rituximab – a placebo-controlled study for psychosis   Meaning:  a. I consent to data about me being processed in the manner described in the research subject information.  b. I agree that the study monitor have access to medical records for checking the data.  c. I agree that the research group have access to my data. | | ☐ | | ☐ | |
| - I agree to provide samples of spinal fluid through lumbar puncture and that these are saved in the biobank. | | Yes  ☐ | | No  ☐ | |
| - I agree to blood samples being taken and stored in a biobank in the manner described in the research subject information. - I agree to undergo MRI examination in the manner described in the research subject information. | | ☐  ☐ | | ☐  ☐ | |

- I agree that relatives or other persons who know me well can

be interviewed about my state of health and fill out a questionnaire after I receive the treatment. ☐ ☐

- I agree to be interviewed about my experiences of the study

after treatment and answer a short questionnaire. ☐ ☐

……………………………….. ……………………………………..

Location Date

……………………………………… ……………………………………………………………

Study participant's signature Responsible study physician
